# Immunoglobulin response to *Plasmodium falciparum* RESA proteins in uncomplicated and severe malaria

**DOI:** 10.1186/s12936-015-0799-8

**Published:** 2015-07-16

**Authors:** Cyril Badaut, Léa Guyonnet, Jacqueline Milet, Emmanuelle Renard, Rémy Durand, Firmine Viwami, Gratien Sagbo, Francis Layla, Philippe Deloron, Serge Bonnefoy, Florence Migot-Nabias

**Affiliations:** Equipe résidente de recherche en infectiologie tropicale, Institut de Recherche Biomédicale des Armées (IRBA), Brétigny sur Orge, France; Institut de Recherche pour le Développement, UMR 216 Mère et enfant face aux infections tropicales, Paris, France; COMUE Sorbonne Paris Cité, Faculté des Sciences Pharmaceutiques et Biologiques, Université Paris Descartes, Paris, France; Laboratoire de Parasitologie-Mycologie, Hôpital Avicenne, AP-HP, Bobigny, France; Centre d’Étude et de Recherche sur le Paludisme Associé à la Grossesse et l’Enfance (CERPAGE), Cotonou, Benin; Service de Pédiatrie, Centre National Hospitalier et Universitaire Hubert K. Maga, Cotonou, Benin; Unité de Biologie Cellulaire des Trypanosomes, Institut Pasteur, INSERM U1201, Paris, France; Faculté de Pharmacie, IRD UMR216, 4 avenue de l’Observatoire, 75006 Paris, France; Paris Cardiovascular Centre (PARCC), Institut National de la Santé et de la Recherche Médicale (INSERM) U970, Université Paris Descartes, Sorbonne Paris Cité, Paris, France

**Keywords:** *Plasmodium falciparum*, severe malaria, Ring-infected erythrocyte surface antigen, B cell epitope, Benin

## Abstract

**Background:**

The three members of the ring-infected erythrocyte surface antigen (RESA) proteins family share high sequence homologies, which impair the detection and assignment to one or another protein of some pathogenic processes inherent to *Plasmodium falciparum* malaria. The present study was intended to determine if the antibody and inflammatory responses of children living in a malaria-endemic area varied depending on the RESA-1, RESA-2 or RESA-3 proteins and the severity of the disease, two groups of severe and uncomplicated malaria cases being considered.

**Methods:**

Two synthetic peptides representing predicted B cell epitopes were designed per RESA protein, all located outside of the 3′ and 5′ repetition blocks, in order to allow an antibody detection specific of each member of the family. Recombinant rRESA-1B and rRESA-3B proteins were also engineered. Two groups of Beninese children admitted to hospital in 2009 for either uncomplicated or severe malaria were compared for their plasma levels of IgG specifically recognizing each recombinant RESA protein or synthetic peptide, and for their plasma inflammatory cytokine levels (IFN-γ, TNF-α and IL-10), taking into account host and parasite genetic factors.

**Results:**

The absence of IgG cross-reactivity between rRESA proteins and their protein carrier as well as between each RESA peptide and a non-epitopic RESA control peptide validated the use of the engineered recombinant proteins and peptides for the measurement of plasma IgG. Taking into account age, fever duration and parasitaemia, a multiple logistic regression performed on children clustered according to their antibody responses’ profiles concluded to an increased risk of severe malaria for P2 (representative of RESA-1) responders (*P* = 0.007). Increased IL-10 plasma levels were found in children harbouring multiclonal *P.* *falciparum* infections on the basis of the T1526G *resa2* gene polymorphism (*P* = 0.004).

**Conclusions:**

This study provided novel tools to dissect the seroreactivity against the three members of the RESA protein family and to describe its relation to protection against malaria. It suggested the measurement of plasma antibodies raised against specific peptides to serve as predictive immunologic markers for disease severity. Lastly, it reinforced previous observations linking the T1526G *resa2* gene mutation to severe malaria.

**Electronic supplementary material:**

The online version of this article (doi:10.1186/s12936-015-0799-8) contains supplementary material, which is available to authorized users.

## Background

Although estimated incidence rates of malaria have declined by 30% globally between 2000 and 2013, and mortality rates by 47%, malaria is still the most widespread of all human infectious diseases, responsible for an estimated 5,84,000 deaths in 2013 [1]. Despite the observation of the largest absolute decreases in deaths in Africa, the disease still affects especially this continent and particularly sub-Saharan Africa.

*Plasmodium falciparum* is the most prevalent (80% of all infections) and lethal (90% of deaths occurring) of the malaria parasites infecting humans. Malaria pathogenesis is linked to the erythrocytic cycle of the parasite. Immediately after the red cell invasion by the parasite, trafficking of hundreds of *P.* *falciparum* proteins to the erythrocyte cytoplasm and membrane gives rise to a progressive mechanical, functional and antigenic remodelling of the host cell in order to create an adequate environment and to overcome host responses. One such protein, called Pf155/RESA (ring-infected erythrocyte surface antigen, RESA-1), stored within dense granules in the invasive merozoites, is released in the parasitophorous vacuole upon invasion and exported to the erythrocyte membrane very shortly after invasion [2], where it interacts with the erythrocyte cytoskeleton protein spectrin [3], stabilizing the infected red blood cell cytoskeleton [4] and conferring increased erythrocyte membrane rigidity upon febrile exposure [5–7]. RESA-1 is the best-known protein of a small protein family encoded by three highly related genes (PFA0110W *resa1*; PF11_0511 *resa2* and PF11_0509 *resa3*). RESA-1 and RESA-3 show a high sequence homology including a PEXEL motif known to be important for the appropriate trafficking of many *Plasmodium* exported proteins [8]. Both RESA-1 and RESA-3 have two repetitive domains (referred to as bloc 1 and bloc 2 repeat domains) and a domain with a high homology to the human chaperone protein DnaJ [9]. Although slightly polymorphic, a peptide domain sharing homologies with the RESA-1 spectrin-binding domain is found on RESA-3. In contrast, RESA-2 does not contain these two repetitive domains nor display any homology with the spectrin-binding domain of RESA-1. The *resa2* gene was initially described as a pseudogene [10] based on the presence of an internal stop codon, supposed to be deleterious, at position 1526. However, another study showed that *resa2* is expressed in the parasite [11]. In some cases, the restoration of a complete protein, thanks to a mutation, occurs and this non-truncated protein could be related to the physiopathology of severe malaria [12].

In this study, the main goal was to determine if the immune response of children living in a malaria-endemic area varied depending on the protein (RESA-1, RESA-2 or RESA-3) and the severity of the disease: uncomplicated malaria (UM) or severe malaria (SM). For this purpose, a transversal survey was conducted in the CNHU of Cotonou, Benin, among a population of 102 children including 54 affected by SM and 48 by UM.

As previously done with the DBL6ε domain of VAR2CSA [13, 14], two peptides representing different predicted B cell epitopes from each RESA protein were used. Plasma immunoglobulin (Ig) G directed to peptides from RESA-1, -2 and -3 as well as RESA-1 and -3 recombinant proteins were evaluated by ELISA. RESA-1 has long been shown to be targeted by the adaptive immune response in populations living in endemic areas: antibodies reacting with RESA-1 inhibit erythrocyte invasion [15–18] and are associated with protection against clinical malaria [19–23]. The immunological response to RESA-2 and RESA-3 proteins is still unknown. Furthermore, many serological studies used synthetic peptides corresponding to C-terminal EENV repeats [16, 20, 23–25] or non-repetitive RESA-1 peptides [17, 26, 27], all shared with RESA-3 and, therefore, making uncertain the true antigenic specificity of the immune response and its functional relevance. Plasma levels of pro-inflammatory (TNF-α and IFN-γ) and anti-inflammatory (IL-10) cytokines known to be involved in both pathogenesis and defence mechanisms against malaria [28–32] were also measured. The red blood cell genetic polymorphisms resulting at the sixth amino acid position of the β chain of haemoglobin (Hb) in the replacement of a glutamic acid by a valine (HbS) or a lysine (HbC) are known to impact the immune response [33–36] and to be protective against malarial attacks [37–39]. Considering their importance in West Africa, [40, 41] their prevalence rates were determined in the population.

Except for RESA-1, the antigenic characteristics and functions of the RESA proteins family remain quite unknown. This study was an opportunity to continue the exploration of the different functions and implication of this proteins family. It allowed namely to dissect the immunological response (in terms of seroreactivity and plasma inflammatory cytokines) against RESA family during a plasmodial infection, according to disease severity. It also offered the opportunity to investigate the role of a recently described mutation occurring in the *resa2* gene.

## Methods

### Study population and sampling

At the arrival of the children in the paediatrics department of the CNHU, gender, age, place of residence, anti-malarial drug intake, and duration of symptoms prior to enrolment were documented by questionnaire. After clinical examination by a paediatrician, observations concerning the ongoing malarial attack were recorded. Patients and samples were previously described [12]. Briefly, 102 children with symptomatic *P.* *falciparum* malaria were recruited in the CNHU of Cotonou, Benin, from April to August 2009. They all lived in the urban area of Cotonou where malaria transmission is heterogeneous according to areas but perennial, with two seasonal peaks corresponding to rainy seasons, from April to July and September to November [42]. The first group (n = 54) corresponded to children admitted to the Intensive Care Unit for SM. All presented at least one of the symptoms defined by the World Health Organization as criteria for SM (severe anaemia, altered consciousness, convulsions, hypoglycaemia, acidosis, respiratory distress, and impaired visceral functions). The second group consisted of 48 outpatients with an UM attack. For each individual a 5 mL venous blood was collected in vacutainers containing citrate phosphate dextrose adenine anticoagulant, before drug treatment administration. Plasmas were stored at −20°C for subsequent antibody testing. Infection with *P.* *falciparum* was identified through the use of rapid diagnostic tests (Parascreen^®^, Zephyr Biomedical Systems, Goa, India) and peripheral parasitaemia by microscopy on Giemsa-stained blood smears. In addition, for each individual, two drops of fresh blood were collected onto filter paper for molecular studies. Negative control plasmas were obtained from 18 healthy European adults who had not been exposed to malaria (Bichat-Claude Bernard Hospital, Paris, France). A pool of four plasmas from malaria immune Gabonese adult donors was used as a positive control.

The study was approved by the ethics committee of the Faculté des Sciences de la Santé of the University of Abomey-Calavi in Benin. For each child, a written informed consent from parents or legal guardians was obtained. The study was conducted in accordance with the Declaration of Helsinki.

### Antigens

#### Synthetic peptides from RESA-1, RESA-2 and RESA-3 proteins

**Figure 1:**
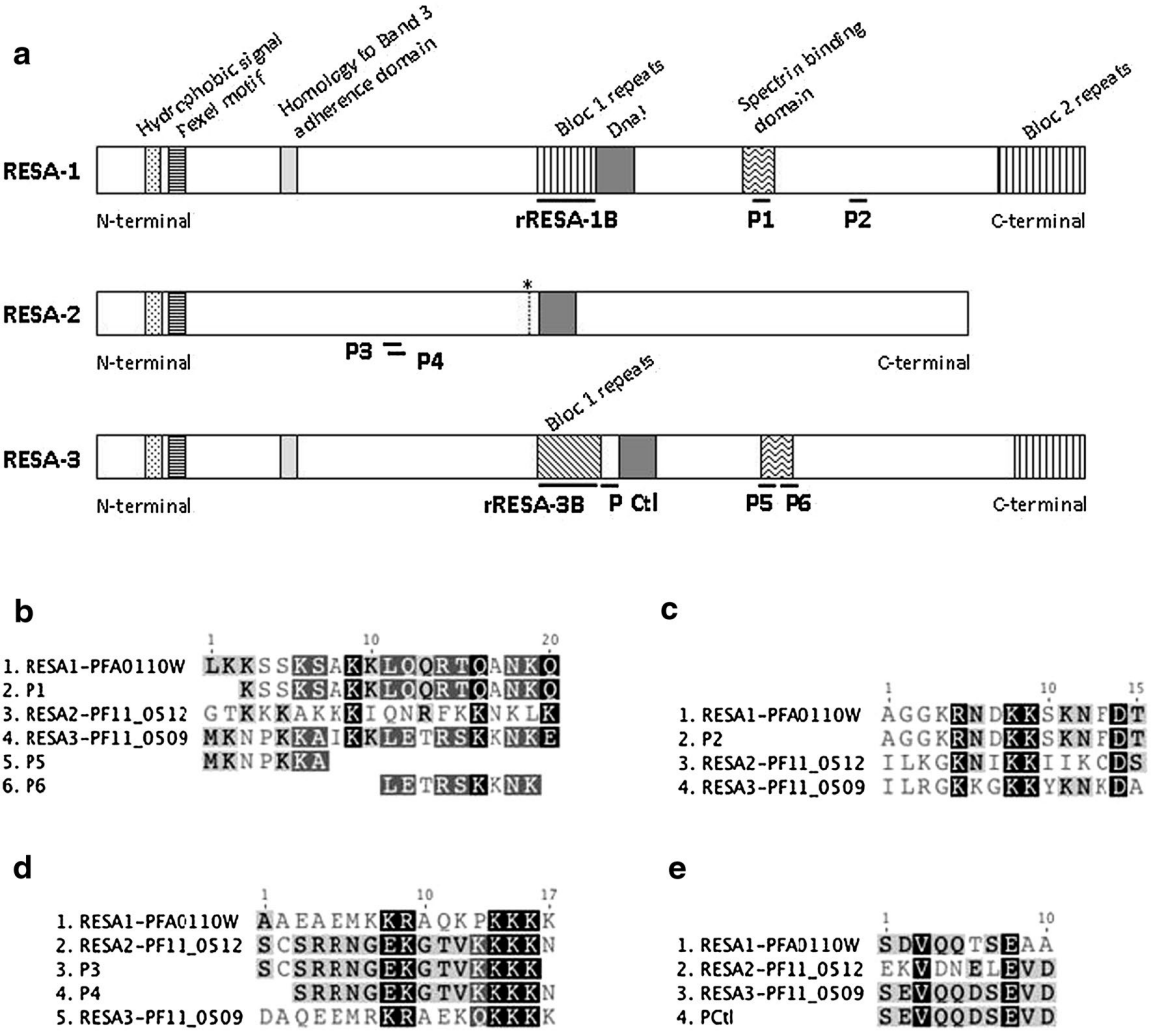
Schematic map of the location of RESA peptides and recombinant proteins on RESA protein sequences. RESA-1 is encoded by the gene *PFA0110w*, RESA-2 by *PF11_0512* and RESA-3 by *PF11_0509*. The schematic representation of the three RESA proteins (**a**) is detailed at the amino acid level for the regions containing peptides P1, P5 and P6 (**b**), P2 (**c**), P3 and P4 (**d**) as well as P Ctl (**e**). * internal stop codon

RESA family proteins share high homology sequences. Protein sequence alignments were performed with RESA-1 (accession number: PFA0110W/PF3D7_0102200), RESA-2 (accession number: PF11_0512/PF3D7_1149500) and RESA-3 (accession number: PF11_0509/PF3D7_1149200) using the Geneious software 6.1.4. from Biomatters Ltd, by means of the integrated multiple **s**equence **c**omparison by **l**og-**e**xpectation (Muscle) algorithm and the default settings. RESA-1 and -2 (see Additional file 1) shared 38% (333 amino-acids) homology, RESA-1 and -3, 49.1% (535 amino-acids) and RESA-2 and -3, 38.9% (361 amino-acids). Thus, cross-reactive antibodies should exist, preventing specific detection of the antigenic protein. To perform ELISA to evaluate the presence of RESA specific antibodies, six synthetic peptides that reproduced putative B cell epitopes of the RESA proteins were used. These peptides, whose sequences are shown in Table 1, were identified following B cell epitope predictions [43]. They were chosen to be located outside of the C-terminal and central repeats, which contain already known Pf155/RESA (RESA-1) epitopes [44], and to allow detection of protein specific antibodies. More precisely, they were chosen to be on a location where sequences are not conserved across the RESA proteins, leading to a given RESA protein-specific antibody detection. These peptides will be subsequently referred to as P1–P6, P1 and P2 being located on RESA-1, P3 and P4 on RESA-2, and P5 and P6 on RESA-3, as illustrated in Figure 1a. The peptides P1 (Figure 1b) and P2 (Figure 1c) shared 100% homology with RESA-1 and respectively, 11.1% (2 aa) and 26.7% (4 aa) homology with RESA-2, and 38.9% (7 aa) and 33.3% (5 aa) homology with RESA-3. The peptides P3 and P4 (Figure 1d), homologous to RESA-2, shared respectively 26.3% (5 aa) and 20% (3 aa) homology with both RESA-1 and -3. The peptides P5 (Figure 1b) homologous to RESA-3 shared no homology with RESA-1 or RESA-2 and P6 share 44.4% (4 aa) with RESA-1. A seventh peptide located on RESA-3 and not corresponding to a B cell epitope was assigned as control peptide (P Ctl) for antibody measurement. P Ctl (Figure 1e) was 60, 40 and 100% homologous to RESA-1 -2 and -3, respectively. The peptides were synthesized with an N-terminal biotin group. They were solubilized and used at a final concentration of 0.3 µM.

**Table 1:** Characteristics of synthetic peptides from the RESA proteins family

Antigens	Sequence	Residues	Abbreviation
RESA-1	Biotin-KSSKSAKKLQQRTQANKQ-COOH	715–732	P1
	Biotin-AGGKRNDKKSKNFDT-COOH	845–859	P2
RESA-2	Biotin-AEKSCSRRNGEKGTVKKKK-COOH	317–335	P3
	Biotin-SRRNGEKGTVKKKKN-COOH	322–336	P4
RESA-3	Biotin-MKNPKKA-COOH	763–769	P5
	Biotin-LETRSKKNK-COOH	773–781	P6
	Biotin-SEVQQDSEVD-COOH	555–564	P Ctl

#### Recombinant proteins from RESA-1 and RESA-3

Divergent central repetitive domains of RESA-1 and RESA-3 proteins were expressed as a carboxy-terminus fusion protein of the maltose binding protein (MBP) recombinant proteins will be referred to as rRESA-1B and rRESA-3B.

Central repetitive domains of RESA-1 (PlasmodB *PFA0110W*, bp 1428-1727) and RESA-3 (PlasmodB *PF11_0509*, bp 1407-1760) from the *P.* *falciparum* FUP/CB strain were amplified using 5′-GGATCCATGTTAGATACATCTGAAG-3′ (sense) and 5′-CTCGAGTTAAACATCACTAGCTGGTTC-3′ (anti-sense) as well as 5′-GGATCCGATGGATCTGAAGCG-3′ (sense) and 5′-CTGCAGTTAAACATCACTAGCTGGTTC-3′ (anti-sense) primers, respectively. The resulting PCR fragments were flanked by *BamH1* and *Pst*I sites, which allowed, following confirmation of PCR products and digestion with both enzymes, cloning in frame into the corresponding restriction sites of the pMal-c4X polylinker.

rRESA-1B and rRESA-3B maltose-binding fusion proteins were overexpressed in XL10 Gold ultracompetent cells (Agilent). Overnight cultures were diluted 1/100 in Luria broth (LB) medium containing ampicillin (100 μg ml^−1^) and bacteria were grown until the A_600_ reached ~0.5. Induction of MBP fusion proteins was induced with Isopropyl ß-D-thiogalactoside (IPTG) at 0.1 mM final concentration. After 2 h at 37°C, bacteria were harvested by centrifugation, and the pellet was resuspended cold buffer (20 mM phosphate buffer pH8, 200 mM NaCl, 0.1% triton X100) supplemented with protease inhibitors (Complete EDTA free, Roche) and benzonase. Lysis was completed by freezing at −20°C overnight and thawing. Following centrifugation at 9,000*g* for 30 min at 4°C, cleared supernatant was collected. Soluble rRESA-1B and rRESA-3B MBP recombinant proteins purification was done using same buffer equilibrated Amylose resin column (New England Biolabs). rRESA-1B and rRESA-3B MBP recombinant proteins were eluted using this column buffer supplemented with 10 mM maltose. Collected fractions (3 mL) were analysed by SDS-PAGE using criterion gels (4–12%). Protein containing fractions were pooled and stored at −20°C.

### Antibody measurements

ELISA was performed to determine plasma IgG directed to the two recombinant proteins and to the six peptides.

Ninety-six-well ELISA plates were coated overnight with 100 µL of recombinant protein solutions as well as MBP protein (New England BioLabs SAS, Evry, France) at final concentrations of 4 µg mL^−1^ and 0.5 µg mL^−1^ in 1× phosphate buffer saline (PBS), respectively. Blocking buffer (1× PBS—3% milk powder—0.1% Tween 20) was added (150 µL per well) and plates were kept at room temperature for 1 h. Regarding the peptides, plates coated with streptavidin were incubated with 200 µL of blocking buffer (1× PBS—0.5% milk powder—0.1% Tween 20) at 4°C overnight and washed three times (1× PBS—0.04% Tween 20). A volume of 100 µL containing each peptide diluted at a final concentration of 0.3 µM in 1× PBS—0.5% milk powder—0.04% Tween 20 was incubated for 1 h at room temperature followed by three washing steps. For both experiments, 100 µL of plasma samples diluted 1:100 (recombinant proteins and MBP) and 1:200 (peptides) in 1× PBS—0.5% milk powder—0.04% Tween 20 were incubated in duplicate for 90 min at 18°C. After three washing steps, peroxidase-conjugated goat anti-human IgG (Fc specific) antibody (A0170, Sigma Aldrich, St-Quentin-Fallavier, France) diluted 1:3,000 for recombinant proteins and 1:3,500 for RESA peptides, was incubated for 1 h at room temperature. Bound enzyme was detected with 100 µL TMB (4380L, Kem-En-Tec Diagnostics A/S, Taastrup, Denmark), the reaction stopped with 30 µl of 0.25 M sulfuric acid and the optical density (OD) was read at 450 nm (reference filter 550 nm).

Reference positive (PC) and negative (NC) pooled control plasmas were present in each plate so as to enable calculation for each plate and each peptide of a normalization factor for inter-plate comparisons. These normalization factors were established according to the formula ([ODm PC] − [ODm NC])/([OD PC] − [OD NC]) where ODm is the mean reactivity of PC or NC for all plates for a defined peptide and OD PC or NC is the mean reactivity of the control plasma samples for a defined peptide of a defined plate. The thresholds for positivity were determined from the mean OD reactivity + 2 SD of 18 plasma samples from non-immune individuals and were 0.741 (MBP), 0.137 (rRESA-1B), 0.000 (rRESA-3B), 0.026 (P Ctl), 0.387 (P1), 0.459 (P2), 0.487 (P3), 0.482 (P4), 0.185 (P5), and 0.352 (P6).

### Cytokine assays

Plasma samples were assayed in duplicate for IFN-γ, TNF-α and IL-10 using ELISA assay according to the manufacturer’s instructions (Mabtech, Stockholm, Sweden). Results were expressed in pg mL^−1^ by reference to standard curves prepared in each plate with recombinant cytokines. Thresholds of sensitivity were 2 pg mL^−1^ for IFN-γ, 13 pg mL^−1^ for TNF-α and 0.5 pg mL^−1^ for IL-10. Zero was assigned to values below the thresholds.

### DNA genotyping by PCR–RFLP

Human and parasite DNA was extracted from blood spots as described previously [12].

#### Determination of the parasite *resa2* T1526G mutation

The presence of the T1526G mutation was detected by *MseI* RFLP. A short *resa2* gene fragment containing the target base was first amplified by PCR using 5′-TGATGCCGTAAAAGATGGTG-3′ (sense) and 5′-TCATATCTGCATTTATATCGACACCT-3′ (anti-sense) primers. Then enzymatic digestion of the PCR products by *MseI* (New England BioLabs SAS, Evry, France) left uncut those fragments carrying the T1526G single nucleotide polymorphism [12].

#### Determination of the human HbS and HbC haemoglobin abnormalities

Carriage of HbS or HbC results from a single mutation on the exon 6 of the beta globin gene, located on chromosome 11. For HbS, an A to T substitution leads to a modification of a glutamic acid into a valine at position 6 whereas for HbC, a G to A substitution leads to a modification of a glutamic acid into a lysine at the same amino acid (aa) position. The same 5′-AGTCAGGGCAGAGCCATCTA-3′ (sense) and 5′-CAGCATCAGGAGTGGACA-3′ (anti-sense) primers were used for the determination of both mutations by PCR amplification of a 369 bp product, on which RFLP was performed, using enzymatic digestion of DNA by *DdeI* and *BseRI* (Ozyme, St-Quentin-en-Yvelines, France) for HbS and HbC, respectively. Both mutations abolished the enzyme restriction sites. The amplified beta globin DNA subjected to *DdeI* cleaved into three fragments (201, 93 and 75 bp) when Hb normal (HbA) and into two fragments (294 and 75 bp) when HbS. When subjected to *BseRI*, it cleaved into two fragments (259 and 110 bp) when Hb normal (HbA) and remained intact (369 bp) when HbC.

### Statistical analysis

#### Effects of clinical co-factors on severity of malaria

Clinical data collected at the time of admission and genetic data were compared between the SM and UM groups. Differences in means were tested by non-parametric Mann–Whitney *U* test or Kruskal–Wallis test (for more than two groups to be compared), except for age, where the Student’s unpaired *t* test was used as age was normally distributed.

A multiple logistic regression was performed to test simultaneously the association between these factors and the severity of malaria. Starting with a complete model including all factors, a stepwise procedure with a backward selection identified the most relevant predictors. The procedure based on the Akaike information criterion (AIC) was implemented in R software. Quantitative independent variables (age, axillary temperature, fever duration, and parasitaemia) were categorized to check if they were linearly related to the log odds. Only age (in year) was found linearly related to the log of odds and was included as quantitative variable in the model. The three others were considered as categorical: three categories were defined for duration of fever (1–2, 3–4 and ≥5 days) and axillary temperature (<38, 38–39.5 and ≥39.5°C); parasitaemia were divided in quartiles.

#### Analysis of the anti-RESA antibody response

The relationships between immune responses to RESA and the severity of malaria were explored in two different ways. First, a non-hierarchical cluster analysis was performed to identify groups of children with similar immune responses, and study the relation between these groups and the risk of severe malaria. Second, the same approach as for clinical factors was used through a multiple logistic regression with a backward selection to identify the set of antibody responses associated with the severity of malaria.

Children were grouped into distinct clusters on the basis of their similarities of immune responses to RESA peptides and recombinant proteins independently of their clinical status. The k-means method implemented in Cluster 3.0 software [45] was used to partition children on the basis of their qualitative responses (responder or non-responder) to the eight antigens. K-means algorithm aimed to find the best partition of n entities in k clusters (where k is user-defined) so that the total distance between the group members and its centroid is minimized [46]. Each child was defined by an 8-dimensional vector of binary data (one dimension for each antigen). The similarities between children were measured by the Euclidean which is defined as:

$$d\left( {x,y} \right) = \frac{1}{n}\sum\limits_{i = 1}^{n} {\left( {x_{i} - y_{i} } \right)^{2} }$$

where *x*_*i*_ and *y*_*i*_ represent the response values for children *x* and *y* respectively against antigen *i*, and n is the total number of antigens considered. K-means cluster analysis was successively performed for different numbers of groups (k = 3, 4 and 5). As partition obtained by this method depends on initial random assignment, for a given k, 2,000 iterations of K-means clustering algorithm were run to ensure to get the optimal clustering solution. At the end the best partition was chosen considering the number of times the optimal solution was found among the 2,000 iterations and examining the different partitions of children. For partitions with k > 3, k-means algorithm identified small groups of children with less than ten individuals, thus a three-group partition was retained. Visualization of results was done using Java TreeView software [47]. The relation between the clusters and clinical status was tested including the groups in a multiple logistic regression adjusted on factors that will be found associated with the severity of malaria.

In the second analysis, a multiple logistic regression was performed with a backward procedure. The initial model included all the antibody responses (considered as binary variables) and clinical factors associated with SM.

#### Analysis of the cytokine patterns

Pearson correlation was calculated between quantitative antibody responses and cytokine production. Mann–Whitney *U* test or Kruskal–Wallis test were used to compare cytokine production between the SM and UM groups, groups defined by genetic defects (*resa2* T1526G mutation, HbS, HbC) and immunological groups.

For all analyses, due to the large number of statistical tests performed, *P* values less than 0.01 were considered significant.

## Results

### Clinical characteristics of the children

The severe malaria group consisted of 47 children with severe malarial anaemia only, one child with cerebral malaria only, and six children with both severe anaemia and cerebral malaria.

Table 2 summarizes the main characteristics of the SM and UM groups of children. The sex ratios of the SM and UM groups did not differ (*P* = 0.63). UM children were older than SM ones (*P* < 0.0001). At admission, UM children were more febrile than SM children (*P* = 0.01) but their fever tended to last for a shorter time (*P* = 0.07). Patients of the SM group had a trend for higher parasite density than patients of the UM group (*P* = 0.06). The prevalence rate of abnormal haemoglobins was 19.6%, distributing equally between the carriage of HbS (9.8%) and HbC (9.8%), mostly at the heterozygous state. Only one HbSS and one HbCC children were recorded, in the SM and the UM groups, respectively. There was no HbSC carrier. The clinical presentation of malaria was not affected by the type of haemoglobin or by the presence of the T1526G mutation in the *resa2* gene.

**Table 2:** Characteristics of 102 children with severe (SM) or uncomplicated (UM) malaria

	SM (n = 54)	UM (n = 48)	*P*
Sex ratio (M/F)	1.7 (34/20)	1.4 (28/20)	0.63^c^
Age (years): mean (±SD)	2.9 (±1.7)	7.1 (±3.8)	<0.0001^d^
Fever duration (days): median (interquartile range IQ25–75)^a^	4 (3–5)	3 (2–5)	0.07^e^
Axillary temperature (°C): mean ± SD^b^	38.4 (±0.8)	38.9 (±1.0)	0.01^d^
Parasitaemia (%): median (interquartile range IQ25–75)	4.3 (1.1–13.0)	3.0 (1.0–6.0)	0.06^e^
Haemoglobin genotype (n)			
HbAA	46	36	0.41^c^
HbAS or HbSS	4	6	
HbAC or HbCC	4	6	
*resa2* T1526G allele (n)			
W	43	43	0.19^c^
WM	3	3	
M	8	2	

^a^1 missing value.

^b^16 missing values.

^c^ *P* value of the Chi-Square test.

^d^ *P* value of the Student’s unpaired-*t* test.

^e^ *P* value of the Mann–Whitney *U* test.

When clinical (age, temperature, fever duration, and parasitaemia) and genetic (haemoglobin genotypes, *resa2* T1526G alleles) factors were considered in a multiple logistic regression, the final model defined by the backward procedure included the four clinical factors (Table 3). In this model, risk of severe malaria decreased linearly with age (odds ratio (OR) [95% confidence interval (CI)] = 0.35 [0.17; 0.58], *P* = 2.7 × 10^−4^) whereas parasitaemia values distributed in the upper quartile [OR (95% CI) = 6.70 (1.30; 54.90), *P* = 0.04] favoured the occurrence of severe malaria. Both axillary temperature at enrolment and fever duration were kept in the model even if associations were not significant (global *P* = 0.06 and 0.13, respectively). The fever duration (3–4 days compared to 1–2 days) was weakly associated with a higher risk of SM [OR (95% CI) = 5.71 (1.01; 42.95), *P* = 0.06] and a high axillary temperature (≥39.5°C) appeared indicative of an UM [OR (95% CI) = 0.12 (0.01; 0.98), *P* = 0.06]. No impact of haemoglobin genotypes or *resa2* mutation was recorded at this final stage of the analysis. In the following analyses, association between anti-RESA antibody responses and severe malaria were adjusted on age, parasitaemia and fever duration. As axillary temperature could not be considered itself as a predictor of severity of malaria infection it was not included in models in order to avoid over fitting.

**Table 3:** Clinical factors associated with severe malaria

Parameters	N^a^	Odds ratio (95% CI)	*P* ^b^
Age (years)	86	0.35 (0.17; 0.58)	2.7 × 10^−4^
Fever duration (days)			
1–2	18	1	0.13
3–4	41	5.71 (1.01; 42.95)	
≥5	27	2.69 (0.40; 21.93)	
Parasitaemia (quartiles)			
Q1–Q3 (<8%)	71	1	0.04
Q4 (≥8%)	15	6.70 (1.30; 54.90)	
Axillary temperature			
<38°C	23	1	0.06
38–39.5°C	44	1.03 (0.22; 4.47)	
≥39.5°C	19	0.12 (0.01; 0.98)	

^a^Information on axillary temperature was available for 86 children.

^b^Presented is the final logistic regression model obtained by a stepwise procedure based on AIC criterion.

### Anti-RESA antibody responses in relation to clinical malaria

#### Validation by the measured IgG of the design of recombinant proteins and peptides

ELISA performed with a rRESA protein can detect not only antibodies raised against this protein but also against the other RESA proteins. The ELISA assay for the measurement of anti-rRESA IgG was validated by the fact that sera recognized and cross-reacted with both rRESA-1B and rRESA-3B (Spearman rank correlation, ρ = 0.451; *P* = 0.0002) while the same sera did not cross-react with the MBP control protein (for rRESA-1B : ρ = 0.162; *P* = 0.13 and for rRESA-3B : ρ = 0.023; *P* = 0.13).

Because the hypothetic B cell epitopes were chosen in non-homologous parts of each RESA protein, the measurement of anti-peptide antibodies was RESA protein-specific. Similarly, a validation of the ELISA assay for the measurement of anti-peptide IgG was brought by the absence of recognition by sera of the non-epitopic peptide P Ctl, as well as by high variability in the sera recognition of distinct peptides. Plasma IgG directed to P1 and P3, P1 and P4, P3 and P4 as well as P5 and P6 were also highly correlated, either for the whole group or for SM and UM groups considered separately (all ρ ≥ 0.475; all *P* ≤ 0.0005). In the same way, IgG against P2 and P4, P2 and P5 as well as P2 and P6 were highly correlated for the UM group only (all ρ ≥ 0.410; all *P* ≤ 0.005). No correlation was observed between anti-rRESA IgG and IgG directed to the associated peptides.

#### Results from the univariate analysis

**Figure 2:**
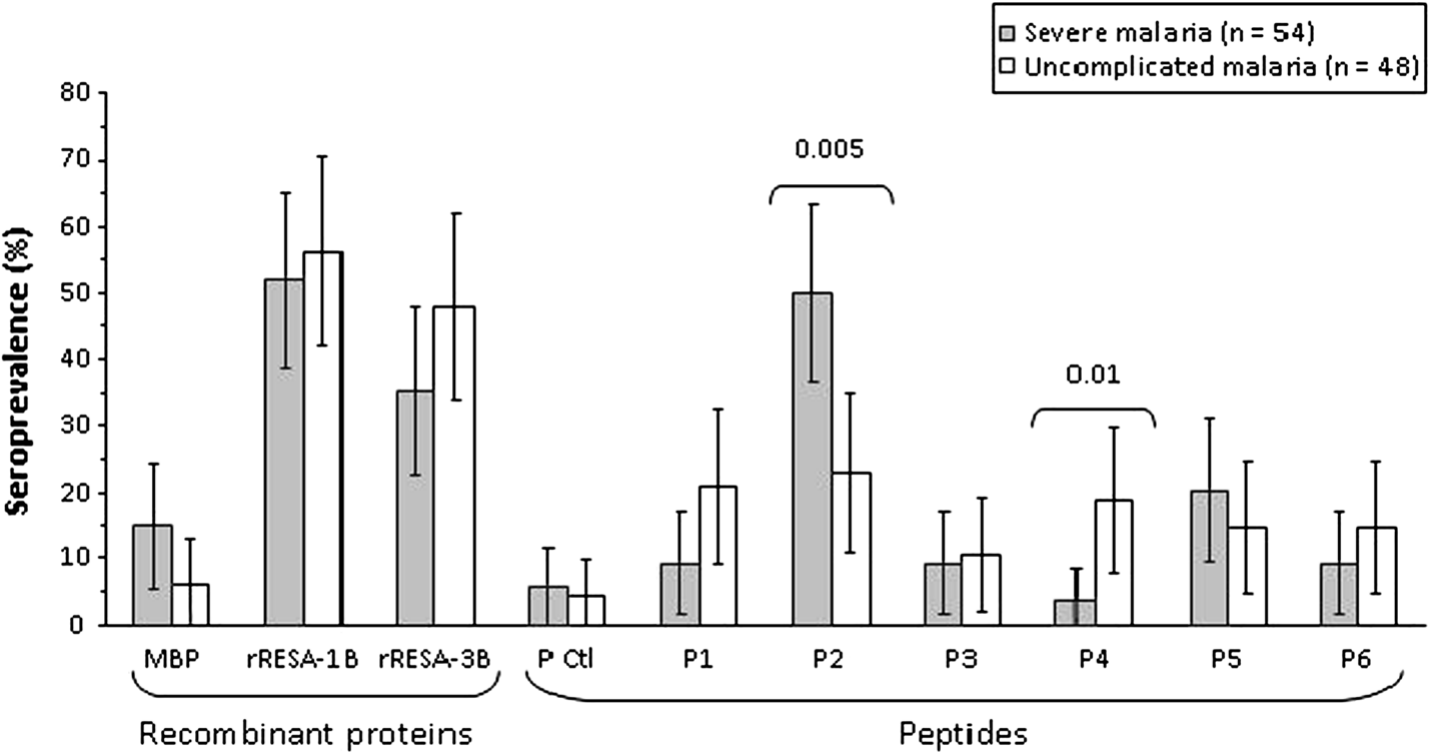
Prevalence rates of plasma IgG to RESA antigens in Beninese children with malaria. *Bars* denote the 95% confidence interval. Significant *P* values of the Chi-Square test (*P* < 0.01) are indicated.

Prevalence rates of IgG responses to RESA antigens according to the clinical presentation of malaria are illustrated in Figure 2, and ranged from 35.2 to 56.3% for responses to RESA recombinant proteins, and from 3.7 to 50.0% for responses to RESA peptides. The highest numbers of responders were recorded for rRESA-1B and one of its representative peptides, P2. Responders to P2 were more numerous among children with SM compared to UM (50.0 vs 22.9%, *P* = 0.005) inversely to what was observed for P4, with 3.7% of responders among SM patients *vs* 18.8% among children with UM (*P* = 0.01). Responders from both clinical groups had similar IgG levels, as presented in Table 4. Presence of abnormal haemoglobin (HbS or HbC) was not associated with any IgG levels to RESA proteins and peptides, except a trend towards lower IgG levels to P2 in HbC (AC or CC genotypes) carriers in comparison to HbAA ones [median OD (IQ25–75) = 0.6 (0.5–0.6) vs 1.0 (0.7–1.4), *P* = 0.02].

**Table 4:** IgG levels to RESA antigens in responder children according to host and parasite characteristics

Group	rRESA-1B	rRESA-3B	P1	P2	P3	P4	P5	P6
SM	0.7 [0.3–1.7] (28)^a^	0.1 [0.1–0.7] (19)	0.7 [0.5–1.0] (5)	0.9 [0.6–1.42] (27)	0.8 [0.7–1.4] (5)	1.2 [1.0–1.5] (2)	0.4 [0.2–0.5] (11)	0.6 [0.5–1.0] (5)
UM	1.1 [0.4–1.9] (27)	0.2 [0.1–0.5] (23)	0.5 [0.4–0.6] (10)	0.8 [0.6–1.1] (11)	0.6 [0.5–0.6] (5)	0.6 [0.5–0.7] (9)	0.2 [0.2–0.3] (7)	0.9 [0.5–1.3] (7)
*P* ^b^	0.28	0.67	0.18	0.81	0.08	0.10	0.22	0.46
HbAA	0.7 [0.3–2.0] (43)	0.2 [0.1–0.5] (31)	0.5 [0.4–0.8] (11)	1.0 [0.7–1.4] (30)	0.7 [0.5–0.7] (8)	0.6 [0.5–0.7] (8)	0.2 [0.2–0.4] (13)	0.6 [0.4–1.1] (11)
HbAS or HbSS	0.7 [0.6–1.1] (9)	0.2 [0.2–0.6] (7)	0.5 [0.5–0.6] (2)	0.5 [0.5–1.0] (3)	1.8 [1.8–1.8] (1)	1.8 [1.8–1.8] (1)	0.3 [0.3–0.4] (4)	(0)
HbAC or HbCC	1.4 [0.8–1.8] (3)	0.5 [0.1–0.9] (4)	0.4 [0.4–0.5] (2)	0.6 [0.5–0.6] (5)	0.6 [0.6–0.6] (1)	0.7 [0.6–0.7] (2)	0.3 [0.3–0.3] (1)	0.9 [0.9–0.9] (1)
*P*	0.61	0.49	0.24	0.02	0.43	0.22	0.66	0.88
*resa2* T1526G allele	0.9 [0.4–2.3] (8)	0.3 [0.1–0.4] (8)	0.4 [0.4–0.4] (3)	0.9 [0.7–1.3] (7)	0.6 [0.6–1.0] (3)	0.5 [0.5–0.5] (1)	0.3 [0.3–0.4] (2)	0.4 [0.4–0.4] (2)
no *resa2* T1526G allele	0.7 [0.3–1.7] (47)	0.2 [0.1–0.8] (34)	0.6 [0.5–0.8] (12)	0.8 [0.6–1.4] (31)	0.7 [0.6–0.8] (7)	0.7 [0.5–0.7] (10)	0.3 [0.2–0.4] (16)	1.0 [0.6–1.1] (10)
*P*	0.45	0.90	0.04	0.95	0.91	0.53	0.57	0.08

^a^Median values [25th–75th percentiles] expressed in OD; (number of responders).

^b^ *P* value for the Mann–Whitney *U* test (two groups) or Kruskal–Wallis test (three groups); significant *P* value < 0.01.

#### Results from the multivariate analysis

**Figure 3:**
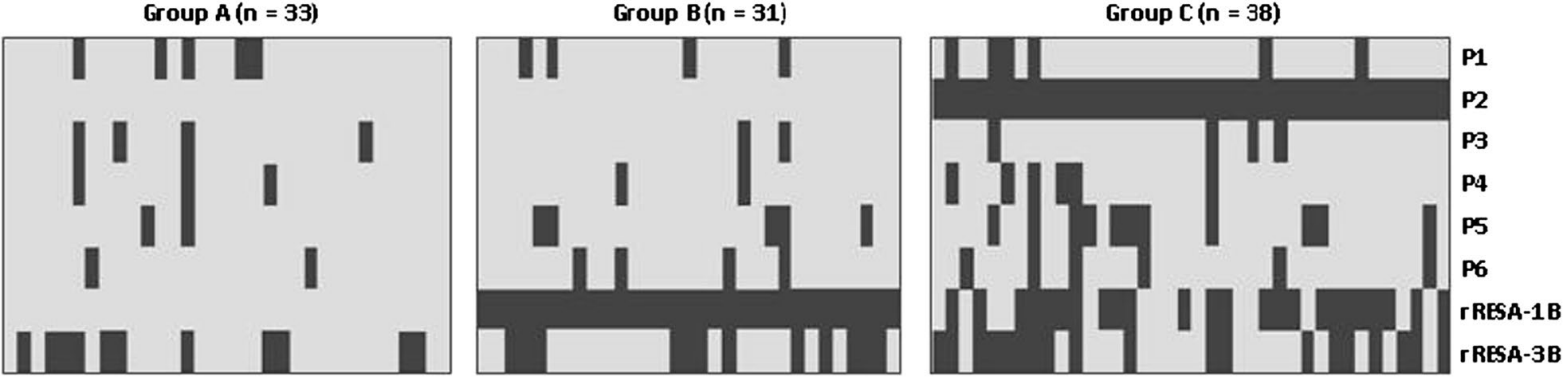
Graphical representation of anti-RESA antibody profiles in three groups obtained by non-hierarchical cluster analysis. Each row represents the children’s responses to one antigen: P1 to P6 synthetic peptides; rRESA-1B and rRESA-3B recombinant proteins. In columns are pictures of the responses against the different antigens for one child. Thus each *rectangle* at the intersection between rows and columns represents the response against one antigen for one child. A *dark grey box* represents a positive response and a *light grey box* a negative one.

The cluster analysis concluded to an optimal partition of children into three groups presenting particular patterns of their anti-RESA antibody responses (Figure 3). Thirty-three children without any defined antibody response profile constituted the Group A. Group B brought together 31 responders to rRESA-1B but not to P2 and Group C gathered 38 responders to P2 who also responded in main cases to rRESA-1B. Severe malaria cases distributed unequally between groups, with 27 cases (50%) in Group C, followed by 16 cases (31%) in Group A and 11 cases (20%) in Group B. When considering Group B as the reference group for investigating the cluster association with SM by a multiple logistic regression adjusted on age, fever duration and parasitaemia, groups were differently related to SM (global *P* = 0.013) with a greater risk for Group C [OR (95% CI) = 7.19 (1.85; 33.37), *P* = 0.007] and a trend for Group A [OR (95% CI) = 4.11 (0.97; 19.76), *P* = 0.06].

In accordance with the results of the cluster analysis, the multiple logistic regression performed with a backward procedure and adjusted on age, fever duration and parasitaemia led to a similar observation, where IgG response to peptide P2 tended to be related to the severity of malaria [OR (95% CI) = 4.10 (1.29; 14.81), *P* = 0.02] (Table 5).

**Table 5:** Association between IgG responses and malaria severity

Antigens	N^a^	Odds ratio (95% CI)	*P* ^b^
P1	15	0.41 (0.08; 1.95)	0.27
P2	38	4.10 (1.29; 14.81)	0.02
P3	10	1.08 (0.16; 8.59)	0.94
P4	11	0.31 (0.03; 2.16)	0.26
P5	18	0.85 (0.16; 4.59)	0.85
P6	12	0.42 (0.05; 4.0)	0.41
rRESA-1B	55	0.43 (0.12; 1.37)	0.16
rRESA-3B	42	0.86 (0.27; 2.78)	0.80

^a^Number of responders.

^b^Each antibody response was tested for association with severe malaria in a multiple logistic regression adjusted on age, fever duration and parasitaemia; significant *P* value < 0.01.

### Plasma cytokine levels in relation to clinical malaria

Plasma levels of inflammatory cytokines (IFN-γ, TNF-α and IL-10) were measured at enrolment in the study and revealed higher TNF-α levels among SM cases than UM ones [median content in pg ml^−1^ (IQ25–75) = 23.5 (0–142.5) vs 0 (0–8.2), *P* = 0.004], as shown in Table 6. Cytokine levels were not associated with IgG positivity to P2 (previously found related to the severity of malaria) nor to rRESA-1B and rRESA-3B, this last antigen being considered in the analysis for the interest placed in the reactivity of the immune system to it. Presence of the *resa2* T1526G mutation was associated with IL-10 plasma levels, the highest cytokine levels being recorded for children with a mixed infection compared to children infected by parasites harbouring only wild type *resa2* T1526G alleles [median content in pg ml^−1^ (IQ25–75) = 98.3 (70.4–176.8) vs 35.2 (17.5–65.1), *P* = 0.004].

**Table 6:** Plasma cytokine levels at enrolment of Beninese children, in relation to host and parasite characteristics

Group (n)	IFN-γ	TNF-α	IL-10
Clinical groups			
SM (54)	10.4 (3.1–30.9)^a^	23.5 (0.0–142.5)	34.2 (16.0–66.9)
UM (48)	5.3 (0.0–39.5)	0.0 (0.0–8.2)	45.9 (24.9–78.0)
*P* ^b^	0.37	0.004	0.28
Antibody responder groups			
Group A (33)	8.3 (2.1–31.3)	0.0 (0.0–88.3)	36.5 (23.7–90.0)
Group B (31)	5.2 (2.1–27.5)	0.0 (0.0–125.4)	43.7 (15.4–64.6)
Group C (38)	10.3 (3.1–33.0)	0.0 (0.0–92.5)	34.2 (14.1–64.3)
*P*	0.70	0.98	0.64
P2—positive response (38)	10.3 (3.1–33.0)	0.0 (0.0–92.5)	34.2 (14.1–64.3)
P2—negative response (64)	6.0 (2.0–31.3)	0.0 (0.0–90.2)	42.2 (19.5–78.0)
*P*	0.42	0.99	0.66
rRESA-1B—positive response (55)	9.9 (3.0–35.1)	0.0 (0.0–109.9)	42.7 (16.9–64.2)
rRESA-1B—negative response (47)	7.4 (2.0–19.6)	0.0 (0.0–69.6)	34.9 (18.7–87.0)
*P*	0.20	0.72	0.99
rRESA-3B—positive response (42)	5.0 (2.0–27.8)	0.0 (0.0 37.9)	39.1 (15.4–63.8)
rRESA-3B—negative response (60)	9.8 (3.0–36.9)	0.0 (0.0–134.1)	38.7 (19.4–72.0)
*P*	0.12	0.09	0.75
HbAA (82)	9.1 (2.4–30.9)	0.0 (0.0–85.1)	41.4 (18.0–79.4)
HbAS or HbSS (10)	12.6 (2.2–36.1)	0.0 (0.0–503.4)	37.2 (16.4–56.8)
HbAC or HbCC (10)	6.5 (2.7–29.8)	0.0 (0.0–60.9)	35.7 (19.1–52.8)
*P*	0.99	0.42	0.69
*resa2* T1526G allele W (86)	9.1 (2.2–29.8)	0.0 (0.00–68.41)	35.2 (17.5–65.1)
*resa2* T1526G allele WM (6)	37.3 (2.7–86.4)	46.0 (15.2–200.3)	98.3 (70.4–176.8)
*resa2* T1526G allele M (10)	9.6 (2.5–34.5)	32.0 (0.0–116.4)	24.7 (12.2–83.5)
*P*	0.68	0.34	0.02

^a^Median values (25th–75th percentiles) expressed in pg/ml^−1^, whole population group considered.

^b^ *P* value for the Mann–Whitney *U* test (two groups) or Kruskal–Wallis test (three groups); significant *P* value < 0.01.

## Discussion

The present study was intended to correlate the presence of immune responses against the three members of the RESA protein family with the severity of malaria attacks. For this purpose, recombinant proteins were engineered for the two repetitive RESA-1 and RESA-3 proteins and peptides representing putative B epitopes of each RESA protein were designed and synthesized. In most previous studies performed on the RESA antigen, peptides were representative of central or C-terminal repetitive RESA-1 sequences [20, 21, 24]. Instead, in the present study, peptides were chosen outside these repetitive blocks in zones of non-homology of the three proteins or corresponding to recombinant proteins reproducing the central and non-homologous repetitive domain of RESA-1 and RESA-3. No corresponding RESA-2 recombinant protein was included as the predicted RESA-2 protein is devoid of such repetitive sequences. These peptides were demonstrated to be the targets of specific antibodies as illustrated by prevalence rates ranging from 3.7 to 50%, which matched anti-peptide antibody prevalence rates reported in previous studies using RESA peptides reproducing parts of the repetitive blocks [25, 27]. Peptides were designed to prevent antibody cross-reactivity within the RESA family.

No correlation was observed between the IgG recognition of rRESAs and synthetic peptides. Since RESA proteins have highly homologous sequences, they should induce cross-reacting antibodies. Thus, the observed IgG reactivity to a defined RESA protein results from a contribution of the IgG responses directed to identical sequences in RESA-1, RESA-2 and RESA-3, but does not reflect the immunogenicity of a defined protein. Moreover, because no RESA three-dimensional structure has been solved, all designed peptides may not have been optimized for their antigenicity and for being fully accessible to the humoral immune system. A single peptide can by no means recapitulate the complete antigenic properties of a full-length protein. Nevertheless, the serum recognition of a pertinent peptide allows estimating the ability of a protein to induce antibodies during the *Plasmodium* life cycle and therefore to relate its presence on the red blood cell surface to the different clinical presentations of malaria. Globally, the antibody responses reported in this study reinforced the interest in the already known immunogenicity of the RESA-1 antigen, as no relation to malaria severity was put forward for antibody responses directed to RESA-2 peptides and RESA-3 peptides or RESA-3 recombinant protein. Indeed, only the anti-P2 IgG response was related to malaria severity. This observation was further supported by a cluster analysis, which allowed distributing children into three groups with distinct profiles of their anti-RESA antibody responses: the group of children responders to P2 and inconsistently responders to rRESA-1B presented an association with malaria severity. This association was independent of age, fever duration and parasitaemia levels.

It was previously shown among the same population group that the T1526G *resa2* gene mutation was associated with parasitaemia >4%, leading to hypothesize that the restoration of a full-length RESA-2 protein could contribute with the other members of the RESA protein family to remodelling the erythrocyte membrane [12] and therefore to favouring high parasite densities which are often associated with the severity of the infection [48]. In this state of mind, it makes sense that high plasma IL-10 levels were preferentially found among patients presenting T1526G mutant parasites. Indeed, IL-10 plays a dual role in malaria pathology: it may be beneficial by reducing the inflammatory response, such as that induced by TNF-α, the plasma levels of which were higher in SM compared to UM children, in agreement with numerous studies [29–31]; however, IL-10 may also be detrimental by decreasing the cellular immune responses which are helpful for parasite control by the host [29]. A subtle imbalance between pro- and anti-inflammatory cytokines determines the course of malaria pathology, and the fact that high TNF-α plasma levels among SM children were not counterbalanced by high IL-10 plasma levels suggests that in SM children at admission, IL-10 had not yet fulfilled its immuno-regulatory role by down regulating the TNF-α inflammatory response [49].

In link with an explanation towards IL-10 as a marker of severity was the observation in the present study of the highest IL-10 plasma levels among children infected by mixed isolates presenting wild and mutant *resa2* alleles. This observation was based on only six samples but still suggests the following hypotheses. Even if the multiplicity of infection (MOI) was not evaluated in this study by genotyping polymorphic parasite genes such as *msp1* or *msp2*, the presence of concomitant wild and mutant *resa2* T1526G alleles was indicative of polyclonality. There seems to be a consensus that high MOI are found during symptomatic malaria episodes of young children from endemic areas, who have a low level of acquired immunity [50]. A study among children in Uganda reported a higher MOI in severe than in uncomplicated malaria [51]. As no association was found in the present study between anti-P3 and anti-P4 IgG, specific of reactivity to RESA-2, and the clinical presentation of malaria, it is not possible to firmly establish whether the restoration of a full-length RESA-2 protein, thanks to the T1526G mutation, may contribute to malaria severity. Such an assessment could be made possible if synthetic peptides, specific of the restored portion of RESA-2, could be designed. Moreover, the above comments would benefit from being strengthened by results of a kinetic study of both cytokine plasma levels and circulating blood parasite clones, which fluctuations cannot be detected by a cross-sectional study.

## Conclusion

To better understand malaria pathogenesis, the role of *P.* *falciparum* proteins exported to the erythrocyte membrane in order to help the infected erythrocyte to escape elimination by the spleen, must be deciphered. It is the case of the three components of the RESA protein family, whose respective roles in increasing red blood cell resistance to febrile conditions are difficult to distinguish due to great sequence homologies. This study offered the first opportunity to dissect the antibody recognition of each RESA protein using synthetic peptides representing specific sequences of each RESA protein. The measurement of naturally acquired IgG in the context of clinical malaria indicated a possible relationship to severe malaria of anti-RESA-1, but not anti-RESA-2 nor anti-RESA-3 IgG. As the design of the study was transversal, the protective role of these IgG cannot be ascertained. Increased IL-10 plasma levels in children harbouring multiclonal *P.* *falciparum* infections on the basis of the T1526G *resa2* gene polymorphism may be suggestive of a pathogenic role of the full-length restored RESA-2 protein. Nevertheless, the small sample size in this study implies the same observations to be made in an independent study or a larger sample in order to be confirmed. At term, the deepening of the understanding of the malaria pathogenesis will provide new possibilities for interventions aimed at reducing parasite survival in the human host.

## Additional file

Additional file 1: Alignment of amino acid sequences of RESA-1, RESA-2 and RESA-3 proteins. Alignment was performed to highlight the homology or the difference of sequences between RESAs and selected peptides.

## Abbreviations

AA: amino acid
AIC: akaike information criterion
CI: confidence interval
CNHU: Centre National Hospitalier Universitaire
ELISA: enzyme-linked immunosorbent assay
Hb: haemoglobin
IgG: immunoglobulin G
MBP: maltose binding protein
MOI: multiplicity of infection
NC: negative control
OD: optical density
OR: odds ratio
PBS: phosphate buffer saline
PC: positive control
RESA: ring-infected erythrocyte surface antigen
SM: severe malaria
UM: uncomplicated malaria
