# Supplementary material for: Immunoglobulin response to Plasmodium falciparum RESA proteins in uncomplicated and severe malaria
Source: Malar J. 2015 Jul 16;14:278. doi: 10.1186/s12936-015-0799-8 (PMC4502540; doi:10.1186/s12936-015-0799-8)

Additional file 1

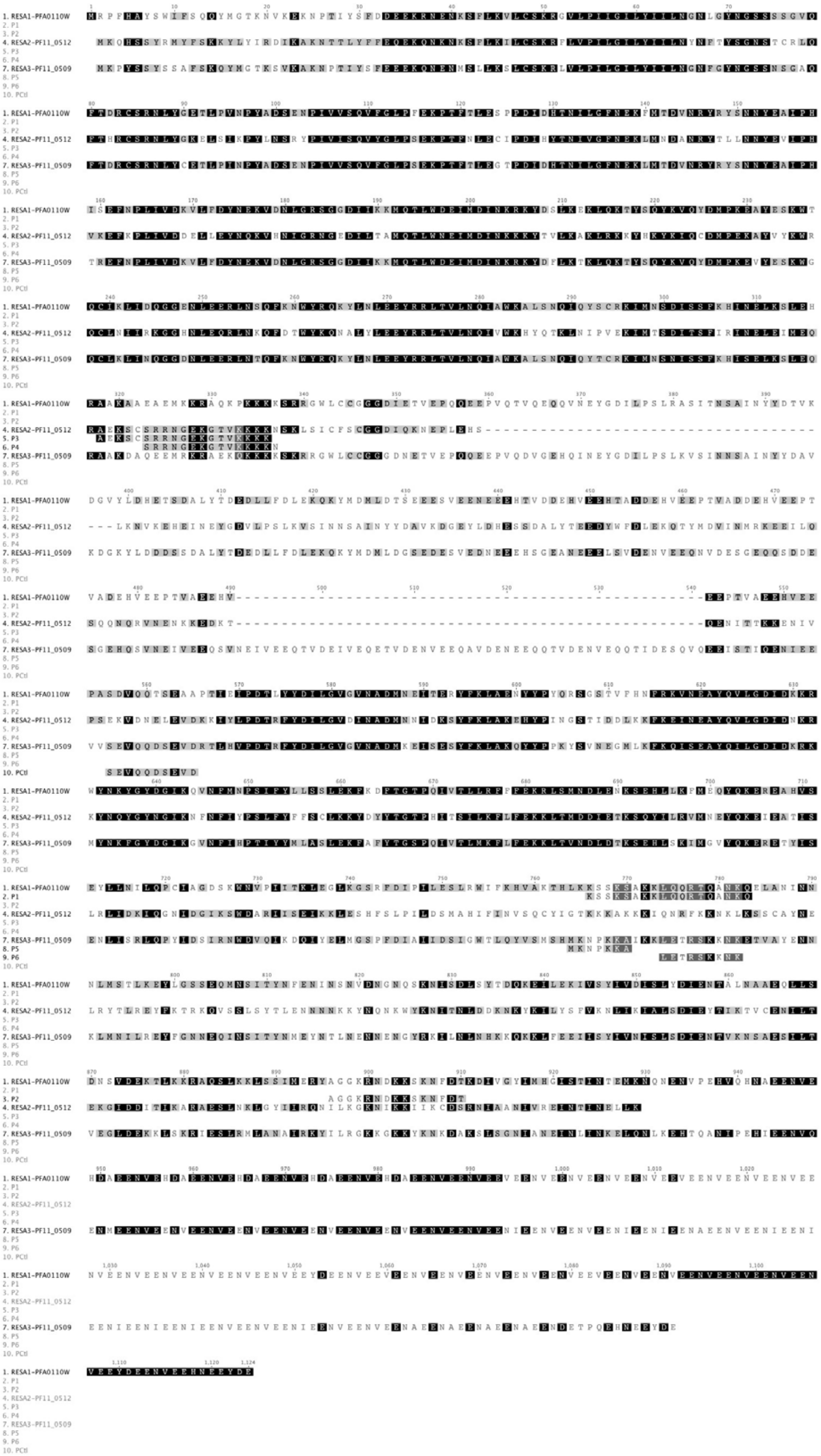

Supplement: Additional file 1: — Alignment of amino acid sequences of RESA-1, RESA-2 and RESA-3 proteins. Alignment was performed to highlight the homology or the difference of sequences between RESAs and selected peptides. [file 12936_2015_799_MOESM1_ESM.pdf]
